# Correction: Microwave-assisted catalytic conversion of glucose to 5-hydroxymethylfurfural using “three dimensional” graphene oxide hybrid catalysts

**DOI:** 10.1039/d0ra90032j

**Published:** 2020-04-14

**Authors:** Yui Hirano, Jorge N. Beltramini, Atsushi Mori, Manami Nakamura, Mohammad Razaul Karim, Yang Kim, Masaaki Nakamura, Shinya Hayami

**Affiliations:** Department of Chemistry, Graduate School of Science and Technology, Kumamoto University 2-39-1 Kurokami, Chuo-ku Kumamoto 860-8555 Japan hayami@kumamoto-u.ac.jp; Centre for Tropical Crops and Bio-Commodities, Queensland University of Technology Brisbane 4000 Australia; Chemistry Department, King Abdulaziz University Jeddah 21589 Saudi Arabia; Department of Chemistry, School of Physical Sciences, Shahjalal University of Science and Technology Sylhet-3114 Bangladesh; Institute of Pulsed Power Science (IPPS), Kumamoto University 2-39-1 Kurokami, Chuo-ku Kumamoto 860-8555 Japan

## Abstract

Correction for ‘Microwave-assisted catalytic conversion of glucose to 5-hydroxymethylfurfural using “three dimensional” graphene oxide hybrid catalysts’ by Yui Hirano, Shinya Hayami *et al.*, *RSC Adv.*, 2020, **10** , 11727–11736.

The Authors regret that an incorrect version of Scheme 2 was included in the original article. The correct version of Scheme 2 is presented below.

**Scheme 2:**
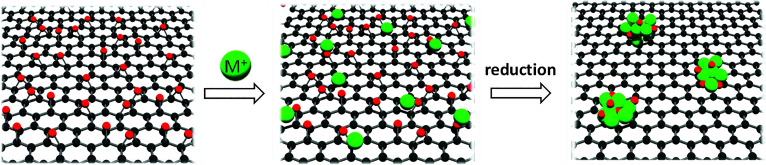
Synthetic strategy for M-rGO.

The Royal Society of Chemistry apologises for these errors and any consequent inconvenience to authors and readers.

## Supplementary Material

